# Structure based functional annotation of a MYND-less lysine methyl transferase in*Candida albicans*

**DOI:** 10.6026/973206300181146

**Published:** 2022-12-31

**Authors:** Joydeb Dey, Himanshu Kishore Prasad

**Affiliations:** 1 Department of Life Science and Bioinformatics, Assam University, Silchar, Assam-788011, India

**Keywords:** SMYD, non-canonical Lysine methyltransferase, Candida albicans, uncharacterized proteins

## Abstract

*Candida albicans* is opportunistic pathogenic yeast that is widely distributed throughout the world and is classified as the most critical fungal pathogen group. *Candida albicans* is a common microbiota of healthy individuals but can cause superficial and invasive infections in immune compromised individuals. Protein Post-translational modifications involving methylation of lysine amino acids stand for a major regulator of eukaryotic transcription, and pathways controlling several cellular processes. SMYD makes up a SET (Su (Var) 3-9, Enhancer-of-zeste and Trithorax) and MYND (Myeloid, Nervy, and DEAF-1) domain containing lysine methyl transferase subfamily that transfers methyl groups from methyl donors onto lysine residues in histones (H3 and H4) and non-histone proteins. The SET domain is the methyltransferase catalytic domain, while MYND participates in both protein and DNA interactions. Well-studied examples of SMYD proteins are five human and two Saccharomyces cerevisiae, constituting examples of histone and non-histone protein lysine methyl transferase members. However, there is limited understanding of SET lysine methyltransferases, including the SMYD subfamily, in the pathogenic fungi *Candida albicans*. Using bioinformatics tools, we characterized the SMYD domain containing proteins in the important pathogen. We report the presence of an atypical SMYD member (CaO19.3863) as a new lysine methyltransferase that can be a target for antifungal therapy.

## Background:

SMYD proteins are a family of lysine methyltransferases that are characterized by a SET and MYND domain. S-Adenosylmethionine (SAM) is a methyl donor that is bound to the SET domain by many lysine N-methyltransferases [1]. The MYND domain is a unique domain that is specific to SMYD proteins, and it is responsible for the recognition of the target lysine residue. The structure of the SET and MYND domains of SMYD proteins is highly conserved among different isoforms, and they are arranged in a characteristic fold that is essential for their enzymatic activity. In particular, the SET domain is composed of an alpha/beta barrel that is stabilized by a conserved zinc ion, while the MYND domain is composed of a helical bundle that is stabilized by two conserved cysteine residues [1,2]. The enzymatic activity of SMYD proteins is mediated by the formation of a catalytic triad comprising the SET domain, the MYND domain, and a lysine residue on the target protein. The SET domain binds to SAM and transfers the methyl group to the lysine residue, while the MYND domain facilitates the recognition and positioning of the target lysine residue [1]. In addition, the MYND domain contains a conserved hydrophobic patch that is thought to interact with the methylated lysine residue. Moreover, the basic residues in MYND domain contribute to DNA binding and the MYND domain of yeast Set5 (Fungal SMYD family) is its mediator in chromatin association and gene repression near telomere [3]. The C-terminal domain of different SMYD members is also involved in protein-protein interactions, localization and control methyltransferase activities in some SMYD members [3]. The five-membered human SMYD family of protein lysine methyltransferases (SMYD1-5) have established roles in muscle immune, blood, heart, vascular endothelial physiology, host-pathogen interaction, and in multiple cancer patho-physiology [4,5]. Their ability to methylate specific lysine residues on histone proteins (K4,36,37 Histones 3 and K5, Histones 4) and hon-histone proteins (HSP90, Rb, P53, MAP3K2) is crucial for regulating gene expression and protein function in cells [4 6]. Overexpression or deregulation of the human SMYD enzymes has been linked to the emergence and progression of cancer, making them promising targets in cancer therapy [5, 7]. SMYD members with structural similarity in Saccharomyces cerevisiae (Set5 and Set6) have similar domain arrangement like their human counterparts. The Set5 enzyme is involved in genome stability and gene expression regulation near telomere mediated by its histone H4 are K5, K8 and K12 methylation activities [3]. In the related opportunistic yeast Candia albicans, the SMYD family of KMT's are relatively uncharacterized, with Candia albicans SET6 expression being regulated by the transcription factor Hap43 and conditions of biofilm and catheters [8]. In this paper, the uncharacterized SMYD family proteins of this yeast was considered for in silico functional and structural characterizations. Using sequence and structure analysis tools, the 3D models were used for identification of residues involved in ligand binding. In this work, we uncover and characterize a novel SMYD member, conserved in the Candida clade organism, which has structural conservation with known SMYD members but completely lacks the MYND zinc finger spanning the SET domain. This protein can be a target for biochemical, genetic,experimental analysis or any future antifungal therapy experiments to ascertain the function.

## Methodology:

## SET domain methyltransferase repertoire of Candida albicans

HMMER 3.1b2 hmmsearch was used to identify all SET domain proteins [9]. Using the Pfam hmm profile (accession PF00856.31), the reference protein database of Candida albicans SC5314 (taxid: 237561) was scanned [9]. The hits were further checked for similarity searches using NCBI BLASTP [10].

## Functional analysis of SMYD Proteins

UniprotKB was used to download well-studied SMYD domain proteins from Human, Mouse, Arabidopsis, toxoplasma, and yeast. As part of InterProscan [11] analysis, these SMYD domain proteins were further functionally annotated by their superfamilies, families,domains, folds, and motifs to identify conserved domains. We also used the HHpred tool for structural annotations using HMM-HMM searches [12]. With ProtParam [13], the physicochemical properties were discovered. Polyphobius was [14] for homology-supported predictions of transmembrane topology, Yloc and DeepLoc 2.0, tool for detecting subcellular localization [15, 16]. In addition,MULocDeep, for suborganelle level localization [17] and SignalP-6.0 [18] to predict signal peptides. DeepTMHMM assigned transmembrane helices [19]. PSIPRED workbench utility, MEMPACK predicted transmembrane helix contact, and DISOPRED3 intrinsic disorders [20]. DeepCoil and Lupas were used to predict coiled-coil domains [21].

## Multiple Sequence Alignment (MSA), Phylogenetic analysis and Conserved Motifs Identification

MSAs were generated with MUSCLE [22], alignments were edited with trimAl in PhyloSuite v1.2.3pre1 [23], substitution model selection and bootstrapped ML phylogenetic tree was constructed using MEGA version 11.0.13 [24]. Conserved sequence motifs were generated using the MEME (Multiple Em for Motif Elicitation) server to predict conserved motif patterns in SMYD proteins [25].

## Secondary structure and other protein features prediction

The PredictProtein webserver was used for features such as protein-protein and protein-DNA binding sites, disorder, and metalbinding sites [26]. PSIPRED 3.2 was employed for secondary structure prediction using neural networks, and CysPRED [27] was employed to detect disulfide bonds. [27]. Protein interactions were discovered through physical and functional correlations using STRING 11.5 [28]. The LambdaPP pipeline [29] was used to annotate gene ontology (GO), binding residues, secondary structure, and variant effect scores.

## Tertiary structure determination and Binding sites predictions

Colabfold notebook for AlphaFold V2 [30] [31] was used to determine the 3D structures of SMYD proteins. Intradomain confidence was derived from predicted LDDT (pLDDT), while domain confidence was derived from a Predicted Aligned Error (PAE) [31]. With AlphaFill [32], ligands, metal ions, and cofactors, information was fitted to high-ranking AlphaFold models. FoldSeek was used for comparative structural analysis [33]. Molviewer was used for visualizations [34]. GalaxyWEB [35] docking tool GalaxySite was used for ligand prediction. The Computed Atlas of Surface Topography of Proteins was used for active site determinations [36]. LambdaPP and Predictprotein were used to predict catalytic and SAM, polypeptide, and metal binding sites.

## Results and discussion:

The SET domain [Su (var) 3-9, zeste enhancer, Trithorax] containing methyltransferases (KMT) of the Candida albicans proteome was identified by sensitive hmmsearch using the hmm profile for SET domain. This search strategy gave eight distinct SET domains containing KMTs (Table 1 -see PDF).

The hits were exhaustively searched by blastp based homology against Uniprot, PDB and nonredundant databases. There are five SETs and three SMYD domain proteins in the Candida albicans genome that constitutes KMT proteins (Table 1 - see PDF). Comparatively, Saccharomyces cerevisiae has only two SMYD domain proteins and five SET proteins (Table 1 - see PDF). In Saccharomyces cerevisiae, Set5 and Set6 are known KMTs of the SET and MYND domains (SMYD) sub-family. Candida albicans have CaSet6 (orf19.1972) and CaSet6 (orf19.3665). This work identified the unique third hit of Candida albicans SMYD KMT (orf19.3836), which has a truncated SET-MYND (SMYD) domain (107 amino acids) and is uncharacterized. The manuscript discusses computational functional and structural characterization of CaSMYD as a novel member of SMYD domain KMT. To identify closely related sequences and estimate its conservation among other SMYD proteins and in budding yeast, the CaSMYD protein sequence was used as a blastp query. The blastp search produced human SMYD3 (14% coverage and 39.13%), mouse SmyD1 (15% coverage and 34.51% identity), human SMYD2 (15% coverage and 35.791% Identity), and AKMT Toxoplasma gondii (21% coverage and 19.71% identity).

Various Arabidopsis thaliana and Schizosaccharomyces pombe SMYD KMTs were found in Uniprot blast (Table 2 - see PDF), however, no orthologs were detected in Saccharomyces cerevisiae or related yeasts in the Saccharomycetaceae family. High-identity sequences were identified among Saccharomycetales through blastp searches. The hits were identified in the genus Candida, Lodderomyces, Spathaspora, Debaryomyces, Scheffersomyces, and Meyerozyma belonging to the CUG-Ser1 clade. Komagataella, Pichiaceae and Saccharomycetales incertae sedis are the other clades having CaSMYD like proteins. Evolutionary analysis using Human, Mouse, yeast, and Toxoplasma gondii proteins identified CaSMYD to be an out group to the Set6 proteins of Saccharomyces cerevisiae and Candia albicans. CaSet5 & ScSet5 clustered with the human SMYD5 protein (Figure 1(A)-see PDF) while CaSMYD, ScSet6, and CaSet6 were out grouped into the human SYMD1-4 cluster (Figure 1(A)-see PDF).

We extended sequence conservation studies with MEME analysis to indicate highly divergent SMYD domains (Table 1 - see PDF). SMYD domain architecture was found in several proteins, including CaSMYD, but with the lowest p-value (Figure 1 (B)-see PDF), suggesting a divergent structure. CaSMYD domains included pre-SET (low identity), SET, and post-SET, domains, but CaSMYD lacked the zinc finger MYND domain C1X2C2X9C3X2C4X5C5X3C6X6H8X3C8 or its variant. The N-terminal ZNF-MYND motif (CX2C, motif of CaSMYD was after the post-SET (FXCXCX2C) (motif#2 Figure.1(B)-see PDF). This was like the ASHR1 (Arabidopsis thaliana) and AKMT (Toxoplasma gondii)(Figure.1 (C)-see PDF) proteins. Indeed, the MEME motif analysis with yeast CaSMYD proteins identified the motif#6 to be C terminal part of a new C2C2 ZNF (CX2CX24CX2C) motif present in closely related Candida species (Figure.1(D)-see PDF). The position of this C2C2 ZNF in CaSMYD spanned amino acid 323 to 372. Also, two unique C-terminal domains (CTD motifs, (Figure.1 (D)-see PDF) were enriched in all non-saccharomyces yeast proteins. According to these results, CaSMYD protein contains SET-MYND domain, but its structure differs from Set5 and Set6 or other SMYD proteins. Therefore, Candia albicans CaSMYD protein contain uncharacterized SET-MYND domain, making it a non-canonical member of the SMYD family of SET MYND sub family of SET lysine methyltransferases. This report presents structural and functional annotations of diverged CaSMYD protein in comparison to other members of this family (Table 3, and 4 - see PDF). This sub-family was present in several yeast clades but not in model organisms S. cerevisiae and Schizosaccharomyces pombe (Figure. 2(A)-see PDF). The molecular weight was predicted to be approximately 73330.87 and reported to be a stable protein with an instability index of 39.90, like CaSet6, while CaSet5 is predicted to be unstable (instability index 50.67) (Table 3 - see PDF ).

A negative gravy value (-0.053) shows that the protein is more water soluble than CaSet5 and Caset6. The globular protein CaSMYD has a higher aliphatic index of 101.94, indicating thermostability over Caset5 and CaSet6. Interpro, SMART, CATH, and CDD databases identified SET domain profiles, but no other domain profiles matched in Candida proteins. Also, the HHpred hmm-hmm profile search yielded the highest CaSMYD hit with human HMT (Smyd1), whereas CaSet5 and CaSet6 profile-profile searches yielded the highest hits with human Smyd2 and Smyd3 (Table 3 - see PDF). The Candida albicans SMYD KMT family comprises canonical CaSet5 & 6 and non-canonical CaSMYD proteins. CaSMYD proteins lack transmembrane domains, coils, or secretory signals, and are predicted to be found both in the nucleus and cytoplasm with a Nuclear Export Signal (NES). CaSMYD amino acids were predicted to bound macromolecules such as DNA, proteins, and small molecules, including metals (Table 3 - see PDF). Based on these findings, it is likely that the reported protein will have similar requirements and function as the other SMYD KMTs, which bind to SAM, zinc, and protein lysine as substrates. It is believed that protein intrinsically disordered regions are crucial structural and functional regulatory regions. CaSMYD was predicted to contain an N-terminal disordered region involved in protein binding. Among the three Candida SMYD methyltransferases, Caset5 was predicted to have a high confidence C-terminal large disorder region (400-473 aa). To increase the confidence of correct functional assignment to the uncharacterized proteins, the protein-protein interaction (PPI) network was evaluated for the CaSMYD protein annotation (Figure 2 (B)-see PDF). Interacting partners of CaSMYD were SET2, SET6, and CTM1 in cluster 1 (PPI enrichment p-value: 6.03e-10). The predicted biological process was methylation (GO:0032259, FDR 0.00045); while the Molecular functions associated was histone-lysine n-methyltransferase (GO:0018024, FDR), Protein-lysine n-methyltransferase activity (GO:0016279, FDR 9.17e-05) and the methyltransferase activity (GO:0008168, FDR 9.17e-05). The predicted KEGG pathway was of lysine degradation (cal00310, FDR 6.98e-08) (Figure 2(B)-see PDF). The secondary structures for amino acid residues were predicted in eight states. Of the CaSMYD protein's secondary structure, 43.02% are helices, 17.94% are extended helices, 33.97% are of random coils, and 5.08% are beta turns (Figure 2 (C)-see PDF). For full-length proteins and domain sequences, AlphafFold machine learning model was used to generate the tertiary structure of SET and MYND proteins. Through CLUSTp 3.0, 108 amino acids were identified as being involved in the formation of the CaSMYD protein active site, based on the best ranked full-length models. The best active site had the Richard�s solvent accessible surface area of 2121.012 Å2 and solvent accessible volume of 1919.608 Å3 (Figure 2(C)-see PDF). We used the AlphFold models to identify homologs using reciprocal best structural hits and sequence similarities. To get the best structural alignment from PDB100 database, we used AlphFold models for three SMYD proteins in PDB files in FoldSeek server search. CaSMYD structure matched the SMYD3 protein with a low Tm score (Table 4 - see PDF), suggesting considerable divergence. We used the generated 3D models to determine the small molecule binding propensity of Candida SMYD KMTs. GalaxySite and AlphaFill webservers predicted the same ligand binding spectrum as the CaSMYD proteins for two canonical Candida SMYD proteins.

Among different predicted CaSMYD ligands were, a cofactor S-adenosylmethionine (SAM), substrate lysine, and metal ions like zinc and nickel (Figure 3(B)(C)-see PDF). Furthermore, we also identified binding of multiple inhibitors of human SMYD2 and SMYD3 proteins bound to the CaSMYD active sites and structural elements. Molecules like sinefungin, 62X, NH5, and LLY-507, are predicted to bind CaSMYD (Table 4 - see PDF). Our manuscript describes the SET and MYND zinc finger KMTs of Candida albicans and proposes a new member of the Set5 and Set6 SMYD subfamily of methyltransferases. This SMYD member lacks a MYND zinc finger. Thus, we have identified a new lysine methyltransferase within the pathogen's genome utilizing well-established and recent tools for annotation of uncharacterized enzymes. We previously reported a Komagataella phaffii ortholog with the SMYD family of yeast [37]. The MYND-less subfamily of SMYD KMTs has been proposed based on analysis of phylogeny, conservation of the SET motif and MYND motif, binding residue prediction, and similarities between 3D structures and ligand binding data. As a result of this study, a solid foundation has been laid for future research into the biochemistry and molecular function of CaSMYD protein in Candida albicans.

## Conclusion:

This study provides an understanding of Candida albicans SMYD methyltransferases, including CaSMYD (orf19.3863), a new member of the family. Protein sequence analysis, structural modelling, and structure-based docking studies were used to determine the potential role of this uncharacterized protein as a member of the protein lysine methyltransferase family.

## ConclusionFunding:

This work was not supported by any financial assistance.
